# That’s the Sex, Baby, and There’s Nothing You Can Do about It! †

**DOI:** 10.3390/metabo11050291

**Published:** 2021-05-01

**Authors:** Nicola De Maria, Erica Villa

**Affiliations:** Gastroenterology Department, University of Modena, 41124 Modena, Italy; demaria.nicola@aou.mo.it

Dr. Bernardine Healy was a cardiologist known, among many other remarkable things, for being the first woman nominated as Director of the NIH. In 1991, Dr. Healy, who died prematurely in 2011, wrote an editorial for *The New England Journal of Medicine* commenting on two studies on sex bias in the management of coronary heart diseases, published on the same journal issue [1]. Its title was “The Yentl Syndrome”, referring to the protagonist of an Isaac Singer story who pretended to be a man in order to study the Talmud. The two studies showed that women were less likely to undergo coronary angiography and surgery as compared with men after being admitted to the hospital with symptoms suspected for myocardial ischemia [2,3]. This was due mainly to the fact that coronary heart disease was thought to be a “predominantly male” disease. Interestingly, women who had proven coronary disease following coronary catheterization had the same likelihood of undergoing angioplasty or surgery as men. As more unsurprising evidence that “women have all too often been treated less than equally in social relations, political endeavors, business, education, and health care” [1], the two studies were a clear demonstration of the Yentl Syndrome: once a woman shows that she is just like a man (in this case, by having a myocardial infarction), then she is treated as a man would be. It is a matter of fact that until 1993, no woman (not even a female rat!) was allowed to be included in studies on experimental drugs by pharmaceutical companies and research centers. 

After 30 years, where are we now? After the Fourth World Conference on Women organized by the United Nations in 1995, we had to wait until the new millennium to have WHO adopting gender-oriented policies and until 2009 to have a written strategy to enhance, expand and institutionalize WHO’s capacity to analyze the role of gender and sex in health, as well as to monitor and address systemic and avoidable gender-based inequalities in health [4]. As stated in the 2009 WHO document, “In order to ensure that women and men of all ages have equal access to opportunities for achieving their full health potential and health equity, the health sector needs to recognize that they differ in terms of both sex and gender. Because of social (gender) and biological (sex) differences, women and men face different health risks, experience different responses from health systems, and their health-seeking behavior, and health outcomes differ” [4].

However, of course, healthcare inequalities are only a part of the major problem of gender-based inequalities. In 2006, the World Economic Forum started publishing the Global Gender Gap Report annually, expressing an index designed to measure gender equality throughout the world. The Global Gender Gap Index has the intention of tracking progress on gaps between women and men in health, education, economics and politics. Across the four categories, the largest gender disparity is in the political field, the second being in economics. To date, in the whole world, only 25% of political seats are occupied by women and only 21% of ministers are women; in some countries, women are not represented at all. In addition, in the last 50 years, in 56% of the countries examined in the report, there has never been a female head of state, including in our country, Italy, or in the United States [5]. As for economy, worldwide, in both private and public corporations 36% of senior managers are women; women’s participation in the job market has been stalling over the last years and, on average, only 55% of adult women are in the labor market, versus 78% of men [5]. Furthermore, in many countries, women are significantly disadvantaged in accessing credit, land or financial products, which prevents opportunities for them to start a company or make a living by managing assets. As for education, gaps are relatively small on average but there are still countries where investment in women’s talent is insufficient [5]. Ten percent of girls between 15 and 24 years of age in the world are illiterate, with a high concentration in developing countries. The sub-index where the average gender gap is the smallest is healthcare, where 95.7% of the global gap has been closed so far. Forty-eight countries have achieved near-parity and many other countries have closed at least 97% of the gap. Yet parity has not been achieved so far, and this means that millions of women around the world are not granted the same access to health as men. Projecting current trends into the future, the overall global gender gap will close in 99.5 years, on average, across the 107 countries covered continuously since the first edition of the report, and we are not going to see this closure happening. 

In the 22 August 2020 issue of the *Lancet*, Dr. Mauvais-Jarvis and his co-authors discussed how women differ from men in medicine [6]. Heart, kidney, liver and pulmonary diseases; cancers; diabetes; infections; neurological and psychiatric diseases are reviewed, and differences between sexes are clearly highlighted. However, as the authors said, “the medical establishment has not assimilated current evidence of sex differences, and the influence of sex and gender on human health and disease continues to be estimated, understudied, and underutilized in medical practice” [6]. This has to do with biases in medical practice, research processes and mainly in medical education. As reported by the authors, male physicians are more effective at treating female patients with myocardial infarction when they work with female colleagues and when they are already experienced in treating female patients. We have been stuck for ages with the belief of Vesalio, the XVIth century anatomist who, studying the human organs, said that everything found about the male body can be applied also to the female body. Certainly, Vesalio did not know the existence of DNA and the fact that the presence of a double X chromosome instead of a Y can profoundly affect not only the susceptibility to diseases but also their clinical presentation. Gene expression differs between the two sexes, with genes being more often overexpressed in women than in men throughout life through the presence of the double X. Then there are hormonal differences, and the blending of all the genetic and hormonal differences ends up in two very different biological systems. This is well known for the immune system, since female sex is generally associated with a stronger immunological response. If that circumstance is of help when dealing with infections or neoplastic cells, it leads to a greater susceptibility toward autoimmune diseases or in cases of disorders triggered by exogenous antigens. However, the biology of women has also a unique feature that differentiates it from men’s, because it changes with the age, being radically affected by the postmenopausal events. The protective effect of female sex hormones dramatically drops with menopause, when the benefit of the anti-inflammatory actions on endothelial and immune cells given by estrogens comes to an end. We can see this generally in all the organs, and also in those not considered a classical target for sex hormones. After menopause, for example, there is a rapid onset or a rapid worsening of the metabolic syndrome, leading to increased intra-abdominal fat, atherogenic lipid profile, steatosis and insulin resistance, and postmenopausal women rapidly lose their advantage over men regarding the risk for cardiovascular events. Our group has investigated, in the last 30 years, the effects of estrogens on the liver, another non-classical target for sex hormones. Male sex is usually predominant in all liver diseases, with the exception of those recognizing a possible immune-mediated pathogenesis, such as PBC and autoimmune hepatitis. If we consider chronic viral hepatitis, several data in the literature show that women of reproductive age have a more favorable natural history than men, with a lower grade of inflammation and slower progression toward severe fibrosis [7]. Again, this advantage is lost with menopause, and postmenopausal women have a higher grade of inflammation and accelerated progression of fibrosis [8]. The same happens also for the leading cause of chronic liver diseases, non-alcoholic steato-hepatitis (NASH). Women of reproductive age are protected from non-alcoholic fatty liver disease, with a ~50% decreased risk compared with men. However, postmenopausal women lose this protection, and premature menopause is associated with a higher risk of non-alcoholic fatty liver disease and related complications among women. The relationship is so strong that the effect of ovarian senescence is evident also in an experimental model of a high-calorie diet in zebrafish [9].

All these are the reasons why NIH in the 1990s decided that women’s health was a priority, and one of the first acts was to fund a multidisciplinary, multi-institute intervention study, the Women’s Health Initiative, addressed to identifying the major causes of death, disability and frailty among middle-aged and older women. However, as we said before, 30 years have passed and yet we still have the feeling that a lot needs to be done before equity between genders/sexes can be achieved. Perhaps the new political course in the United States, with a very determined, professionally proud academic First Lady and an equally determined female Vice-President, will finally give the decisive push to change the scenario.
